# Methylenetetrahydrofolate Reductase Polymorphism and Premature Coronary Artery Disease

**DOI:** 10.7759/cureus.5014

**Published:** 2019-06-27

**Authors:** Ahmed Zaghloul, Corina Iorgoveanu, Aakash Desai, Kathir Balakumaran, Kai Chen

**Affiliations:** 1 Internal Medicine, University of Connecticut Health Center, Farmington, USA; 2 Cardiology, University of Connecticut Health Center, Farmington, USA

**Keywords:** mthfr, polymorphism, coronary artery disease

## Abstract

Methylenetetrahydrofolate reductase (MTHFR) catalyzes the conversion of 5,10-methylenetetrahydrofolate to 5-methyltetrahydrofolate, a co-substrate for homocysteine re-methylation to methionine. Its deficiency leads to an increased serum level of homocysteine, which is well-known to be associated with premature coronary artery disease (CAD). Our case demonstrates the association of MTHFR polymorphism with premature CAD and myocardial infarction (MI) despite normal homocysteine levels. Screening for MTHFR polymorphisms in addition to homocysteine levels may be considered for patients presenting with premature CAD and a normal lipid profile. Aggressive risk reduction with lifestyle modifications and guideline-driven medical therapy supplementation might be necessary for secondary cardiovascular disease prevention until more specific therapeutic options are available for this subgroup of patients.

## Introduction

Coronary artery disease (CAD), also known as ischemic heart disease, is the leading cause of death in the world, for the past 15 years [1]. According to the World Health Organization (WHO), 15 million people were diagnosed with CAD in 2015 alone. A better understanding of the genetics, pathogenesis, and pathophysiology of CAD is crucial to help with the development of therapeutic strategies and, more importantly, preventive strategies pertaining to lifestyle modifications and medical therapy. An understanding of CAD is crucial in trying to help stem the public health crisis, whether it be understanding the pathophysiology, the lifestyle modalities responsible for its emergence, or the genetic components increasing predisposition to its development. Though methods of reducing the mortality rates of those with CAD such as medications and medical procedures have become much more commonplace, the emphasis of sound healthcare maintenance is prevention. Thus, it is no less important, if not more, to understand how to prevent CAD rather than simply treat it.

CAD is an umbrella term that encompasses numerous conditions, such as stable and unstable angina, myocardial infarction, and sudden cardiac death, however, the mechanism underlying the disease processes is largely the same. CAD arises most commonly as a result of an atherosclerotic process, which includes vascular inflammation, fat and calcium accumulation, increased pro-thrombotic activity, and thickening of the inflicted vessel lining. In the process, the vessel lumen gradually narrows, compromising blood flow and oxygen supply. This can lead to conditions such as stable angina and more severe myocardial infarction and death.

Many of the general risk factors pre-disposing to CAD have been extensively investigated and well-understood. These commonly described risk factors include, but are not limited to, hypertension, diabetes, smoking, obesity, hyperlipidemia, physical inactivity, and family history. Among them, lesser known is family history, which stems from one’s own genetics. Aside from the heritability of CAD that is proposed to be anywhere from 40 to 60 percent [2], there are other known health conditions that can inadvertently lead to CAD. These conditions include familial hypertriglyceridemia, hypercholesterolemia, genetic hypercoagulable illnesses, such as Factor V Leiden (the most common one), and homocysteinemia, amongst others [1,3-6]. Numerous tests have been created to measure markers exposing to these conditions such as lipid panels and activated protein C resistance tests. One commonly tested marker, homocysteine, has become a marker of controversy, as its validity has come under question in recent times.

Homocysteine, a non- protein amino acid produced by the breakdown of methionine in vivo has been well-known as far back as in the 1960s when the autosomal recessive condition known as homocystinuria was first investigated. Homocystinuria affects multiple body systems, resulting in afflictions ranging from psychiatric to musculoskeletal. As the name implies, individuals with this condition experience elevated levels of homocysteine in their urine. However, this name is a bit of a misnomer, as those elevated levels of homocysteine in the urine are simply the excretion of homocysteine that is, more importantly, very elevated in the serum.

Researchers noted an associated between increased serum homocysteine and early coronary events - as early as the third decade of life - mirroring extensive atheroma formation and intravascular thrombosis [1]. Since then, various conditions have been associated with elevated homocysteine levels in the blood, including deficiencies in folic acid, vitamin B6, B12 (all components of the pathway responsible for breaking down methionine), systemic lupus erythematosus, hypothyroidism, psoriasis, kidney disease, and methylenetetrahydrofolate reductase (MTHFR) polymorphisms. Some medications, such as methotrexate, can also cause elevated levels of homocysteine through their blockage of the methionine pathway. Of note, all the medical conditions previously listed have also, in some capacity, been linked to an increased risk of coronary artery disease [7].

But what is the exact relationship between elevated homocysteine and CAD? Classically, the evidence pointed towards homocysteine being somehow responsible for the increase in atherosclerosis and thrombosis. Nevertheless, the exact mechanism continues to be unknown. Studies have shown that in those with elevated homocysteine levels, there is twice as high a risk of CAD [7]. The exact mechanisms underlying the association between elevated homocysteine levels and early CAD are largely speculative, with some authors arguing direct causation while others proposing a bystander role for homocysteine, being the degradation by-product of an already inflamed vessel. However, per the European Society of Cardiology (ESC) guidelines on cardiovascular disease prevention in clinical practice, the circulating or urinary biomarker have either no or only limited value when added to cardiovascular disease (CVD) risk assessment.

## Case presentation

**Figure 1:**
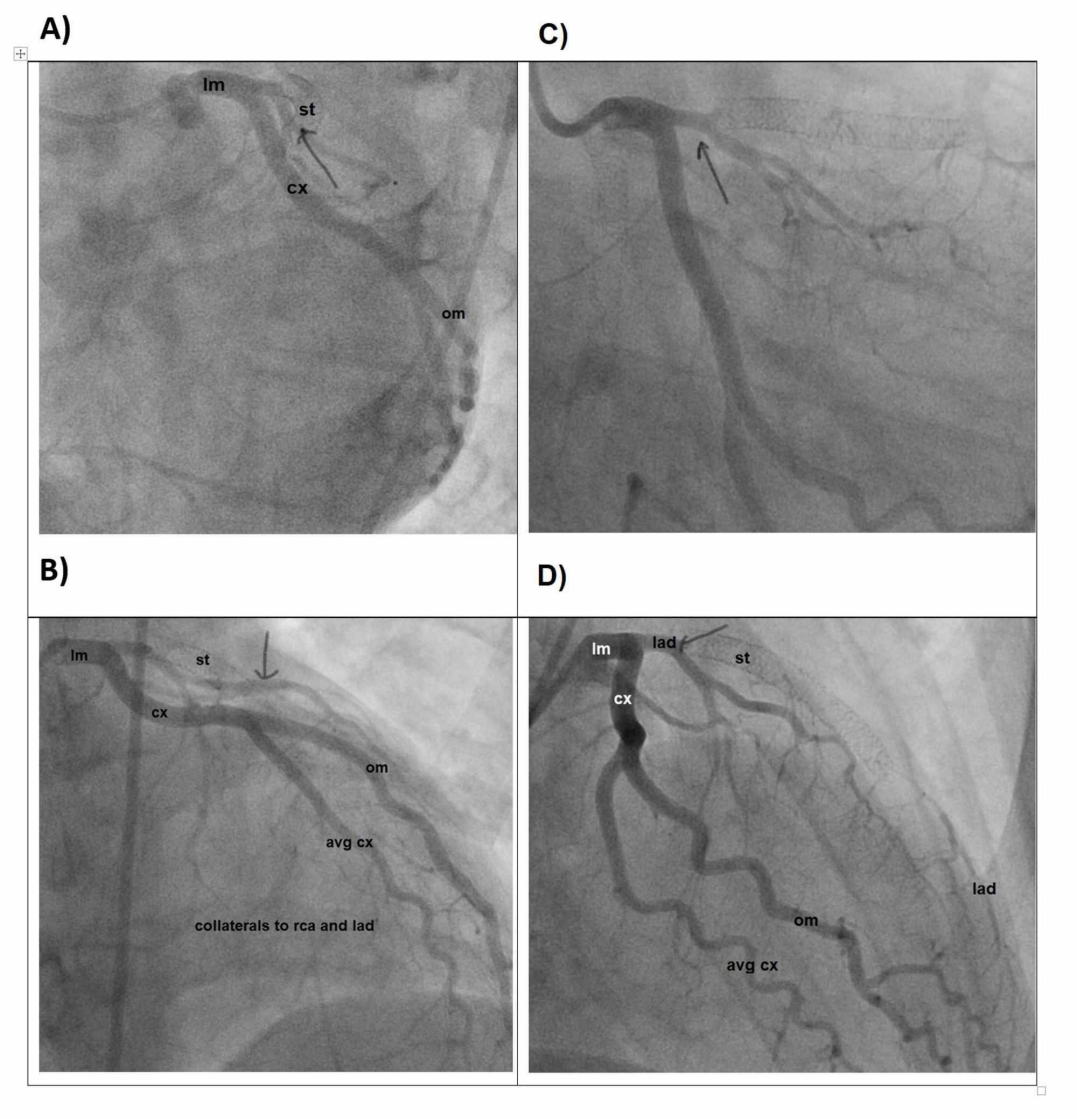
Cardiac catheterization demonstrating the LAD A) LAD in left anterior oblique caudal view; B) LAD in left anterior oblique cranial view; C) LAD in right anterior oblique caudal view; D) LAD in right anterior oblique cranial view cm: Circumflex; om: Obtuse Marginal; st: Stent; lm: Left main; LAD: Left anterior descending

A 23-year-old man with MTHFR polymorphism and a history of an anterior wall ST-segment elevation myocardial infarction (STEMI) requiring drug-eluting stent (DES) deployment in the left anterior descending artery (LAD) at the age of 21 presented to the emergency department (ED) with substernal chest pain, He was found to have T wave inversions in the inferior leads on his electrocardiogram (ECG) and elevated troponin levels. Coronary angiography demonstrated an acute occlusion of the proximal right coronary artery (RCA) and chronic total occlusion of the proximal LAD with collaterals from the circumflex territory (Figure 1).

**Figure 2:**
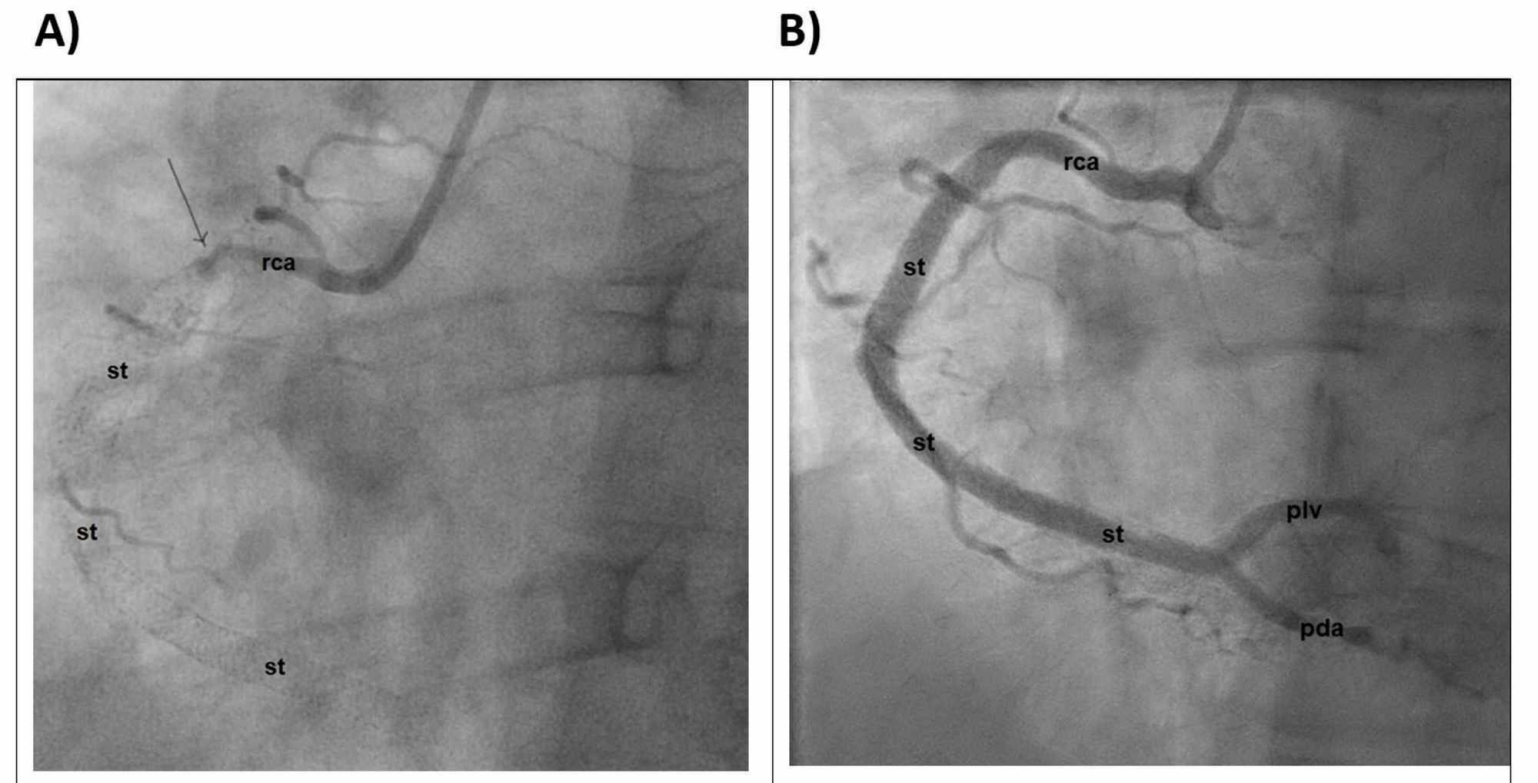
Cardiac catheterization demonstrating the RCA A) Before intervention; B) After intervention st: Stent; pda: Posterior descending artery; piv: Posterior interventricular artery; RCA: right coronary artery

He underwent aspiration thrombectomy of the RCA lesion with three DES placements (Figure 2).

This was followed by continuous infusion of unfractionated heparin and tirofiban for 48 hours in addition to aspirin and ticagrelor. His left ventriculogram showed a mildly reduced left ventricular ejection fraction of 45% with hypokinesis of the basal inferior wall segment.

Hypercoagulability workup was unrevealing. Homocysteine levels were normal at the time of his first myocardial infarction two years prior. The repeat homocysteine level was 11.7 mg/dl (normal cutoff <11.2 mg/dl). His total cholesterol was 153 mg/dl, low density lipoprotein (LDL) cholesterol 80 mg/dl, apolipoprotein B was 67 (normal range: 52-109 mg/dl), lipoprotein (a) <10 (normal <75 nmol/L). MTHFR genotyping confirmed the double-heterozygous C677T/1298C mutation. He was then discharged on folic acid. He was brought back to the cardiac catheterization laboratory a month later to reassess the LAD lesion. The angiogram showed total in-stent occlusion of RCA. The patient was then referred for coronary artery bypass surgery.

## Discussion

We hereby report a patient with MTHFR polymorphism, one of the previously mentioned potential causes of elevated homocysteine and a major risk factor for CAD (Poster: Ahmed Zaghloul, Corina Iorgaveanu. Methylenetetrahydrofolate reductase polymorphism and premature coronary artery disease. Chest; September 10, 2018).

MTHFR is an enzyme that catalyzes the conversion of 5,10-methylenetetrahydrofolate to 5-methyltetrahydrofolate, a co-substrate for homocysteine remethylation to methionine. The gene that codes for the enzyme MTHFR has two alleles. There are currently over 20 gene polymorphisms coding for the enzyme, with the two most studied ones being the C677T and A1298C single nucleotide polymorphisms (SNPs) [8]. While both mutations cause multiple health issues, C677T appears to have the strongest association with CAD. In C677T, the MTHFR nucleotide at position 677 in the gene has a thymine (T) group rather than the wild type cytosine (C) which, in turn, results in a valine amino acid rather than an alanine, with greatly reduced enzymatic activity. Individuals homozygous for the thermolabile enzyme variant have lower MTHFR activity than CC (normal) or even CT (heterozygous) individuals and are much more sensitive to decreased folate levels. Folate is a crucial substrate in the same pathway as the MTHFR enzyme and leads to even higher levels of homocysteine. With homozygous mutation of the C677T type, the outcomes in terms of numerous different conditions are worse since there is only 30% of normal enzyme function. In contrast, with homozygous A1298C, the deficiency is less severe since there is 60% functionality of the MTHFR. The double heterozygous C677T/1298C mutation found in our patient may result in decreased enzyme function. In the literature, no studies detail differences in the severity of cardiovascular events based on various polymorphisms. A study has demonstrated that patients with C677T homozygous mutations have an increased risk of venous thromboembolism (VTE) and thromboembolism (23%) compared to compound heterozygous C677T-A1298C (16%) [5]. Furthermore, Day et al. demonstrated that variants in MTHFR may influence the progression or severity of pulmonary vascular disease [3]. Also, there is a strong association between MTHFRC677T and maternal risk of Down syndrome in Jordanian mothers younger than 35 years old and the MTHFRA1298C allele has a smaller but additive risk effect in MTHFR677T/A1298C compound heterozygotes [9]. These studies suggest that the different polymorphisms in MTHFR mutations can cause variance in disease severity. Given the case at hand, it can be assumed that these polymorphisms may also play a pivotal role in the severity of predisposition to CAD.

In addition, those with the C677T mutations are believed to be at an increased risk for CAD, as well as cancers such as acute lymphoblastic leukemia and colon cancer. It is believed that roughly 10% of the population of North America is T-homozygous for the MTHFR polymorphism with Mediterranean individuals and Hispanics having the greatest frequency, Caucasians having the second highest and those of African origin and African Americans having the lowest prevalence [10].

Classic belief holds that thermolabile (TT) variant MTHFR translates to the highest homocysteine levels and thus most severe CAD. Some works have challenged this notion, showing only a modest risk increment when compared to the CC or CT genotypes [8]. This linear relationship has, however, become a lot less straightforward in recent years, something that parallels the findings in the case of our 23-year-old patient with recurrent coronary events. Though studies have pointed towards those with homozygous thermolabile MTHFR variants being the most at risk for elevated homocysteine levels and ensuing CAD, others such as Naka et. al. have found through their multi-case analysis that homozygous TT individuals were only slightly more likely to be represented amongst patients with CAD and their homocysteine levels to be only slightly higher than those with the CC or CT genotypes. Those who are heterozygotes for the thermolabile polymorphism are roughly as unlikely to have elevated homocysteine findings as are those with the normal non-thermolabile CC variant [8]. While we cannot speak of the TT genotype, our own case does conform to the belief that heterozygous individuals do not experience significantly elevated homocysteine levels. However, our case does present some discordance with the available literature in regard to an elevated homocysteine level being necessary in MTHFR polymorphisms for the occurrence of CAD. Lin et al. found that even in TT variants if their levels of homocysteine are not elevated either naturally or due to treatment with folic acid, there is no reported increased risk of cardiac events over native variants [9]. This again points to homocysteine being the most important factor driving CAD risk in those with MTHFR polymorphisms. Gonzalez-Porras et al. and Vulapalli et al. corroborated this view as the former found that elevated homocysteine levels were a risk factor for recurrent cardiac events in young people regardless of their MTHFR status and the latter demonstrated that MTHFR status has no bearing of its own on reinfarction rates [10-14]. Our case, however, showed the exact opposite as the patient has always had a normal homocysteine level yet has had multiple reinfarctions with the only discernable risk factor for acute coronary events specifically at such a young age, with all hypercoagulability diseases ruled out, being his MTHFR status. This again raises the question of what exactly homocysteine’s role is in the road from MTHFR mutation to acute coronary syndrome (ACS). In our case, the supposed marker of disease is absent, yet the disease itself is overwhelming, completely contradicting the report by Vulapalli et al. [14]. Further complicating the case are the patient’s two older sisters who are also heterozygotes. Their condition has played out much more along the lines of the classical view of heterozygotes for MTHFR: their homocysteine levels are normal and they have not experienced any symptoms of CAD. This suggests the presence of currently unrecognized modifier factors, which may play a crucial role in the mediation of MTHFR status on CAD development. It is important to note that we do not know the body mass index (BMI) of his sisters and his BMI at 35 and his elevated triglycerides, though modestly elevated, may be direct or indirect catalysts for his polymorphism causing CAD at such a young age.

## Conclusions

Further investigations into the relationship between MTHFR genotypes and the incidence of CAD, in particular, re-infarction or re-stenosis, should be based on larger samples, paying attention to the differences between various ethnic populations. It may be that the MTHFR enzyme mutation is part of a bigger constellation of genetic code that, when also mutated, causes the CAD we see. Homocysteine’s role in this disease may be harmful but tangential or maybe it really does fill the role of a marker for something we have not quite yet uncovered. Individual therapeutic strategies based on single nucleotide polymorphism may become increasingly important for preventive treatment against polygenic CAD.
